# Evaluation of the Positive Peers Mobile App for Supporting the Viral Suppression of Young People With HIV: Protocol for a Concurrent Mixed Methods Evaluation With Randomized Controlled Clinical Trial and Observational Cohort

**DOI:** 10.2196/87601

**Published:** 2026-03-17

**Authors:** Mary M Step, L Anthony Catania, Jennifer McMillen Smith, Steven A Lewis, Yanis Bitar, Vinay K Cheruvu, Kristen A Berg, Jeffrey S Hallam, Ann K Avery

**Affiliations:** 1 College of Public Health Kent State University Kent, OH United States; 2 Division of Infectious Disease MetroHealth Medical Center Cleveland, OH United States; 3 Case Western Reserve University School of Medicine at MetroHealth Cleveland, OH United States; 4 Department of Infectious Disease Case Western Reserve University School of Medicine at MetroHealth Cleveland, OH United States

**Keywords:** HIV, young people with HIV, mHealth, HIV support, social support, retention in HIV care, viral suppression, peer support, social media affordances, HIV stigma

## Abstract

**Background:**

People at greatest risk for poor HIV outcomes include young (13-34) people of color who have sex with men. Individuals in this population are least likely to be aware of their HIV status and are at the highest risk for disengaging from medical care and antiretroviral therapy. The Positive Peers mobile app (PPA) was designed to engage this population with real-time social support, HIV and healthy lifestyle information, and medical management tools. We expect that greater PPA engagement will predict key HIV care outcomes. Study predictions are grounded in a user-centric model of digital media use and the perceived affordances of the PPA.

**Objective:**

This study aims to determine the optimal deployment of the PPA in clinical settings (at enrollment vs delayed start) and to compare intervention outcomes with a no-intervention, observation-only condition. The PPA is designed specifically for key HIV disparity populations, including younger sexual, gender, racial, and ethnic minorities.

**Methods:**

The Positive Peers Intervention Trial (PoPIT) is a multisite, randomized clinical trial designed to evaluate the effectiveness of the PPA as a tool for use in clinical settings. Trial arms compare the immediate deployment of the PPA with usual care and observation only. This protocol outlines a mixed methods design consisting of concurrent prospective self-report questionnaires, in-depth interviews with PPA users, and medical record review. PoPIT questionnaires include measures of social determinants of health, HIV-related stigma, perceptions of digital media use, self-efficacy, substance use, and social support. Multiple aspects of PPA intervention engagement are measured natively within the app. Outcomes include HIV National Quality Forum indicators and perceived HIV-related stigma.

**Results:**

The research protocol (1R01MD019185-01) was funded for US $903,363.00 in direct costs by the National Institute for Minority Health and Health Disparities on September 24, 2023, for 5 years. The study began recruiting patients on June 3, 2024, and will continue accrual until November 27, 2026.

**Conclusions:**

Findings will provide evidence of the usefulness of the PPA as a support tool in HIV clinical care. Primary results will inform optimization of PPA deployment and evaluate a theoretical model of user engagement with a mobile health management app. Qualitative data will provide a phenomenological description of intervention engagement and perceived user efficacy.

**Trial Registration:**

ClinicalTrials.gov NCT06388109; https://clinicaltrials.gov/ct2/show/NCT06388109

**International Registered Report Identifier (IRRID):**

DERR1-10.2196/87601

## Introduction

### Background

Younger people under the age of 35 continue to carry a disproportionate burden of HIV risk [1-4]. Within this group, social risk factors such as poverty, drug use, job insecurity, food scarcity, and housing instability present significant barriers to HIV testing, retention in care, and viral suppression [5,6]. These factors contribute to younger people being less likely to be retained in HIV care and to achieve sustained viral suppression, especially those in marginalized communities [1,7]. There is a critical need to establish effective and sustainable tools to address these barriers and improve HIV outcomes for disproportionately impacted young people [4,7].

Several electronic tools have been designed to engage young people with HIV (YPWH) in clinical care and to support medication adherence [8-10]. However, fewer tools link newly diagnosed and out-of-care clinical populations to a live peer cohort that shares the unique challenge of living with HIV [11]. Mobile health (mHealth) apps are gaining credibility as reliable and accessible communication modalities that provide a direct platform for addressing social barriers to adherence, retention, and viral suppression [10,12,13]. Implementation of mHealth interventions is particularly well suited to high-priority jurisdictions where PWH may be socially and geographically isolated from trustworthy information and reliable support [14-16]. The Positive Peers mobile app (PPA) was created in response to these needs as a clinical mHealth tool to help newly diagnosed young people, or those returning to care, connect with each other and learn about living with HIV [17].

### The Positive Peers Mobile App

PPA is a free, multifunction mobile app that features interactive functions organized around a Mind/Body/Spirit theme. The Mind theme is characterized by information provision, including more than 400 vetted articles concerning issues faced by YPWH, as well as first-person stories from current app users living with HIV. The Body theme offers tangible support in the form of interactive medication and laboratory reminders and trackers, and curated state-by-state community resources addressing a variety of living needs (clothing, housing, food). The Spirit theme fosters human connection through a community forum that allows users to ask questions and share experiences and encouragement, as well as a chat function for 1-on-1 conversations. The app is freely available to eligible young people (ages 13-34) who provide evidence of their HIV-positive status. App activity is monitored and supported by a group of trained app administrators at our home medical institution in Cleveland, Ohio, as well as at key partner locations across the United States.

Following development and successful feasibility testing with a pilot group [17], a cohort comparison medical record review of PPA users and clinically eligible but study-naïve nonusers showed that PPA users were significantly more likely to be regularly engaged in HIV care and to achieve higher rates of viral suppression than nonusers, particularly among young adults aged 13-24 [18]. This success, paired with positive reactions identified in qualitative work [19], provided the basis for planning a randomized controlled trial to evaluate the PPA as a clinical tool for supporting YPWH who are newly diagnosed or seeking care in 6 federally designated Ending the Epidemic priority jurisdictions in the United States [14].

The primary objective of the Positive Peers Intervention Trial (PoPIT) is to evaluate the impact of the mHealth app on engagement in care and viral suppression outcomes among newly diagnosed and reengaged young patients. Study research questions and hypotheses are informed by, and evaluated based on, social networking, media uses and effects, and social support theories.

### Social Networking Theory and Social Support

Social Networking Theory (SNT) is an approach to understanding how ideas and behaviors disseminate through relational ties [20]. A key line of research in SNT examines whether and how social networks influence health behavior [21,22]. SNT has guided thinking about information flow and innovation uptake for decades, with more contemporary approaches positioning social network membership as central to identity, community, and health [23-25]. Notably, although several mechanisms of influence have been postulated (eg, modeling, presentation of group norms), the source of behavior change in SNT approaches stems from social ties rather than individual cognition [26-28]. A key mechanism underlying social network effects is social support.

Although social support was initially conceptualized as a dyadic process (see [29]), health-related research has produced ample evidence of the efficacy and benefits of networked social support on various social media platforms, including reduced depression and mitigation of stigma [30-32]. As social media enables a more flexible, collaborative communication process, it offers greater opportunities for engagement with similar others [33-35]. Participants in online support groups have reported strong feelings of empowerment associated with exchanging information, receiving emotional support, experiencing validation and understanding, helping others, and sharing amusement [36,37]. These empowerment processes, in turn, are associated with feeling more informed, experiencing greater social well-being, demonstrating greater efficacy in interactions with physicians and in managing treatment, and greater acceptance of illness [38].

Among PWH, stronger social support has been associated with safer sex [39], better medication adherence [40], fewer disclosure-related concerns [41], lower depression [42], and better overall quality of life [31,43]. Not surprisingly, younger people, and those recently diagnosed with HIV, are more likely to use online rather than face-to-face support, a trend that reverses as they gain experience and begin to trust others [44,45]. Support messaging in this context most frequently takes the form of informational and emotional support [43,45].

Social networking among PWH particularly benefits younger, isolated, or marginalized people who report less in-person support [46]. Stress rooted in chronic illness is known to be associated with a greater prevalence of adverse mental and behavioral health outcomes [47,48]. Ongoing stressors, such as living with HIV, homelessness, or identifying with a population minority, can also motivate social media use and online support seeking [49]. Positive outcomes associated with stress-induced social media use include active coping, the creation of positive group norms, increased self-efficacy, and reduced perceived stigma [37,50-52]. Importantly, unique sources of stress can be mitigated through connection with similarly stigmatized others, particularly within a context of social support and expressed self-compassion [53-56]. The resulting sense of belonging, or social connectedness, generated in these situations can catalyze resilience, increased esteem, and potentially improved self-care [55,57]. These effects are known to be robust even within weak-tie social networks, such as those involving acquaintances [58]. The PPA leverages these processes and effects by providing a secure space where social connections with similar others can aid the management of ongoing chronic illness stressors. The PPA builds on these ideas by using HIV status as a criterion for belonging rather than exclusion, providing secure opportunities for individual or group communication, and disseminating information targeted to network characteristics.

### Digital Media Uses, Effects, and Affordances

The PPA app is informed by 2 user-centric theories of media use: media uses and effects theory and media affordances theory [59-63]. The media uses and effects approach is a model of media use that posits that needs experienced by users are expressed as motives, that is, intentions for media use [64]. Active user motives (eg, information- or support-seeking), rather than passive motives (eg, passing time), are associated with more direct information and affective processing, which further determine the likelihood of cognitive, affective, or behavioral change [65]. This paradigm has been useful for understanding internet use in general and social media effects in particular [61,66]. For example, Phua et al [24] established that social networking site design and interface features were associated with different motives for use and subsequent gratifications received from those sites.

Media affordances theory developed from the media uses and effects approach by accommodating the rapid evolution of technology as a mediator of human communication. Within media affordances theory, specific features, functions, or constraints of social media technology provide opportunities to act relative to a user’s needs or purposes within a given context [67,68]. Media affordances are defined as variable user perceptions of technology features that offer pathways for the gratification of perceived needs [69]. For example, interacting with a peer in the PPA community forum may address a user’s need for social inclusion in a more acceptable way than through a public social media platform, creating a perceived affordance associated with the app. Not all users perceive the same affordances of the same technology. Instead, users’ perceptions of app or device affordances emerge through interaction with that technology. For example, one person may use an mHealth app because it affords a confidential space to connect with similar others, whereas another may be drawn to a comprehensive medication tracking system. These theoretical frameworks suggest that user needs and perceptions of app affordances create a context for seeking gratification from that app that may or may not be reinforced. Experimentation and adaptation shape users’ perceptions of how an app can work for them. These related user-centric approaches to understanding media influence depict a communication process that accommodates variation in user-technology interactions [67,68]. Variability can be found in (1) user characteristics and needs, (2) technology design features, and (3) user-technology interactions and outcomes [68].

### Study Rationale and Objectives

**Figure 1:**
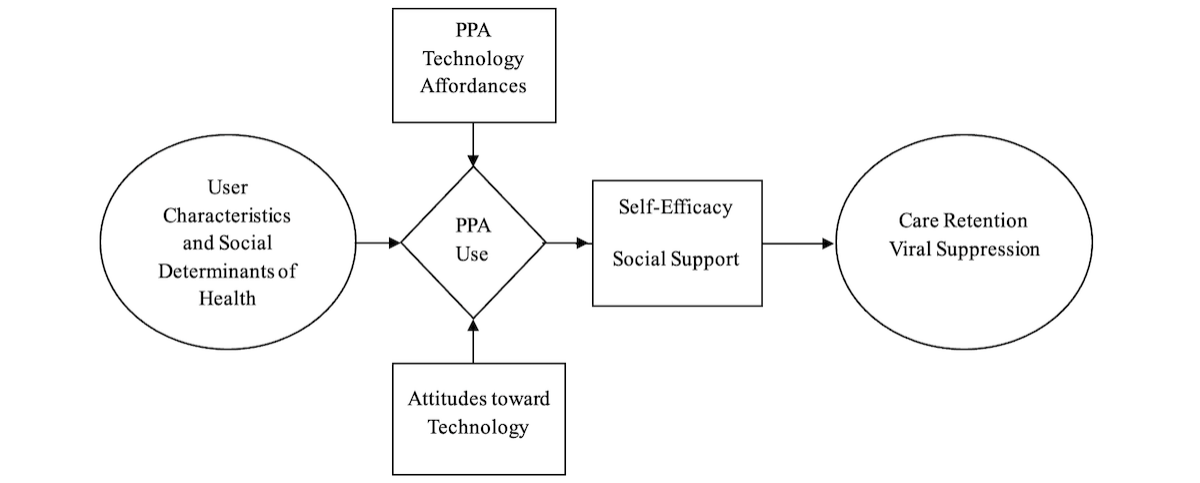
Positive Peers Intervention Trial (PoPIT) study conceptual model. PPA: Positive Peers mobile app.

We expect the PPA to support engagement in HIV care by normalizing and rewarding care visits, promoting health literacy, reducing stigma, and enhancing inclusion, thereby allowing users to help others with similar challenges. Our goal is to increase HIV viral suppression rates by at least 15% by offering practical and peer support through a smartphone app. Specific aims addressed by the protocol described in this manuscript are (1) comparing the effectiveness of HIV care supported by the PPA with usual care for improving retention in HIV care and viral suppression among high-priority adolescents and young adults with HIV; (2) identifying factors that predict user engagement with primary PPA components and associated effects on retention in care, viral suppression, and HIV-related perceived stigma; and (3) evaluating PoPIT implementation across sites and over time. Figure 1 illustrates the conceptual model of the PoPIT study’s influence and outcomes. This paper describes the protocol used to achieve these aims in a randomized clinical trial with a concurrent qualitative evaluation focused on user experience.

## Methods

### Study Design

Specific aims and corresponding hypotheses will be evaluated in a randomized controlled trial that compares HIV outcomes in patient groups randomized to either immediate or delayed access to the PPA. The delayed-start design is intended to ensure that both randomized study conditions receive identical study procedures, follow-up, reminders, incentives, and assessments during the first 6 months of participation. The only difference is the timing of app access. Any improvement observed in the delayed-start group during this period can therefore be attributed primarily to nonspecific effects of trial participation. The primary randomized comparison at 6 months thus estimates the incremental benefit of the app beyond these common trial effects. A greater improvement in outcomes in the immediate-start group at this point would provide evidence of a specific impact of the app.

Prospective questionnaires will be administered at baseline and at 3, 6, 9, and 12 months to provide evidence for model-specific hypotheses and the mediating effects of covariates and demographics. We have included an opportunity for patients who decline full study participation to grant permission to the PoPIT team to abstract their HIV outcomes from medical records on the same schedule as full study participants. This observation-only arm includes only the baseline questionnaire and medical record review of study outcomes. The inclusion of an observational cohort in the randomized controlled trial design strengthens external validity by enabling comparison of trial participants with the broader clinic population. By comparing outcomes from both randomized controlled trial arms with those of the observational cohort, we will gain additional insight into how trial participation may influence outcomes and how the intervention might be further adapted for a more diverse clinical population.

Allocation (1:1) to study conditions is determined by the REDCap system’s randomization module following signed consent and completion of the baseline questionnaire, and before PPA download. Participants are informed of their randomization assignment after completing the baseline questionnaire. If randomized to the immediate intervention condition, site staff assist the participant in downloading the PPA to their personal smartphone and orient them to the app’s functions and study guidelines. Participants randomized to the delayed-start arm receive usual clinic care before returning to the clinic 6 months later to download and begin using the app. This delayed-start cohort serves as a control group, allowing comparison of the effects of intervention exposure with usual care.

The Practical Robust Implementation and Sustainability Model (PRISM) was used to inform intervention implementation throughout the study [70,71] (Table 1). The PRISM model was selected because it accommodates multilevel and dynamic interactions among the intervention, stakeholders’ perspectives, and recipient characteristics. The PRISM model is an expansion of the well-used RE-AIM (ie, Reach, Effectiveness, Adoption, Implementation, and Maintenance) approach, with additional emphasis on sustainability factors both within and external to the study setting [70].

**Table 1:** PRISM^a^-informed implementation plan for the PoPIT^b^ study.

RE-AIM^c^ domain	Before implementation	During implementation	After implementation
Intervention delivery	Estimate population parameters with site staff and set accrual benchmarks. Develop recruitment strategies with site staff.	Log and monitor recruitment and enrollment. Deploy boosting efforts if needed.	Calculate reach and enrollment trajectories.
Effectiveness	Preintervention site visits to determine setting and population characteristics, and resources.	Collect prospective questionnaire data. Medical record review. Conduct user qualitative interviews.	Primary and secondary outcome analysis and qualitative analysis of user interview data.
Adoption	Tailor recruitment materials for sites. Provide clear user support materials and training curriculum for site staff. Create instructional animations for PPA^d^ functions.	Track all admin requests for app assistance, addressing potential barriers as needed. Analyze native app engagement tracking. Review monthly performance trajectories.	Report adoption facilitators and barriers, construct app engagement variables, and track use and attrition trajectories over the project life.
Implementation	Deliver group and individual site trainings to promote fidelity. Design a REDcap^e^ data management system for onboarding and data collection.	Log site administrator app activity and user compliance with data collection. Conduct monthly all-site check-in meetings.	Postimplementation staff interviews.
Maintenance	Develop oversight strategies, meeting agendas, staff interview approach, and questions.	Monthly site staff meetings. Qualitative staff interviews.	Evaluate cost, review systems, identify potential adaptations, and determine dissemination prospects.

^a^PRISM: Practical Robust Implementation and Sustainability Model.

^b^PoPIT: Positive Peers Intervention Trial.

^c^RE-AIM: Reach, Effectiveness, Adoption, Implementation, and Maintenance.

^d^PPA: Positive Peers mobile app.

^e^REDcap: Research Electronic Data Capture.

Implementation planning with PRISM identified a set of evaluation activities before, during, and after the study that provide evidence of intervention exposure, effectiveness, staff adoption of study procedures, and intervention sustainability. A detailed description of the implementation plan and associated outcomes will be reported in a future manuscript.

### Sample Population

The study is designed to evaluate the effects of the PPA intervention among the age group most likely to be newly diagnosed with HIV in the United States: adolescents and young adults. This cohort is challenged by a propensity for greater risk behavior, lower health literacy and management skills, and typically less favorable social determinants of health. Potential participants are identified as patients at 1 of 5 HIV care organizations serving as study sites, located across 6 priority jurisdictions where HIV transmission occurs most frequently. Patients are screened to determine whether they meet all necessary inclusion criteria for study participation (Textbox 1).

**Positive Peers Intervention Trial eligibility criteria.**

1. Inclusion criteria

- HIV-positive status, either newly diagnosed within the last 12 months, out of care (without an office visit in the last 12 months), or not virally suppressed (viral load >200 copies/mL within the last 12 months).
- Adolescents and young adults aged 13-34 receiving care at one of the study sites.
- Identification with a sexual, gender, racial, or ethnic minority.
- Participant’s capacity to provide informed consent (aged >16 years) or assent to participate (aged <16 years).
- Ownership of a smartphone/tablet (either iOS or Android) capable of supporting the Positive Peers mobile app.
- Ability to read English.

1. Exclusion criteria

- Previous use of the Positive Peers mobile app.
- Adults older than age 34 at the time of enrollment.
- Not a patient at one of the participating clinical sites.

### Study Setting

Ending the Epidemic aims to decrease new infections by identifying PWH, increasing access to biomedical and HIV prevention interventions, and improving engagement in care for those already diagnosed [72]. The PoPIT study is being implemented at 6 study sites within high-priority jurisdictions in the United States. Three of these sites are within infectious disease clinics at academic medical centers in Texas, New Jersey, and Washington, while the remaining 3 are located at federally qualified health centers in California and Ohio.

### Recruitment

Eligible persons seeking care or registered with a participating clinic are invited to participate in the study through their preferred contact channel on record with the clinic. They are asked by trained clinic staff at participating sites whether they would be interested in helping to evaluate a new HIV support app in a research study. Persons who agree to participate review the informed consent document with the study staff before any study procedures. Participants can earn up to US $250 in gift cards by completing study questionnaires and interviews. Participants receive US $50 for completing the baseline questionnaire and a qualitative interview, and US $25 for completing each follow-up questionnaire. There is also a monthly raffle for ecological momentary assessment (EMA) completion, in which participants have a chance to win an additional US $25 for completing an EMA questionnaire during that month. If a person declines participation in the randomized trial, they are asked whether they would participate in the more passive observational monitoring arm, which includes a baseline questionnaire and prospective medical record data collection. Participants in the observational cohort are compensated with a US $50 gift card at the time of study consent and completion of the baseline questionnaire.

A reliance review institutional review board (IRB) system is in place, with agreements from all sites to accept the lead study site IRB for regulatory oversight. The general consent form is written in simple, plain language and is devoid of logos and colors. One study site is required to use its own institution’s regulatory language; however, study information is consistent across all sites. The primary investigative team is clearly identified in the consent form, along with corresponding contact information.

### Enrollment, Randomization, and Data Collection Procedures

All participants are consented, enrolled, and randomized at the partner institution where they receive care, in partnership with the PoPIT staff, who are needed to set up the user’s PPA account. Specifically, following in-person consent at the partner site, all participants complete a preloaded baseline questionnaire on a study-dedicated tablet in the presence of study staff. While they are completing the questionnaire, the trial participant is randomly assigned in REDCap to either the intervention (immediate PPA start) or control (6-month delayed PPA start) arm at a 1:1 ratio. Participants randomized to the delayed-start arm receive usual clinic care before beginning the clinical intervention. Given our pilot findings indicating a stronger PPA effect on HIV viral suppression among participants in the youngest age group (ie, <24 years), we employ a block randomization strategy in which study arm assignment occurs separately for participants within the age groups 13-24 and 25-34 years.

Following randomization, site study staff assist participants randomized to the immediate-start arm in downloading the PPA from the public app store using the participant’s personal phone. Site study staff guide participants through in-app tutorials demonstrating how to use app features. Participants randomized to the delayed-start arm are provided with a folder containing general HIV self-management information and contact numbers for the clinic and the site coordinator. Data are collected via online forms (enrollment and medical record review), questionnaires, and SMS text messaging. Participants are consented and enrolled by study staff in person, and the baseline questionnaire is completed independently at the same visit. Remaining follow-up questionnaires at 3, 6, 9, and 12 months are prompted by an SMS text message or email and completed online. Participants and study staff are aware of study arm assignment. Delayed-start participants are given app access after completing the 6-month questionnaire. Site study staff follow the same procedure to assist with downloading the app and reviewing tutorials for participants in both the immediate- and delayed-start arms.

### Intervention

As described above and elsewhere, PPA is a password-protected, multifunction mobile app for supporting retention in HIV care and viral suppression [17]. Before clinical trial use, the color scheme and fonts were refreshed, the digital architecture of the app was evaluated and debugged to ensure reliability, and accessibility was upgraded to comply with Web Content Accessibility Guidelines (WCAG) 2.1 Level AA and Section 508 standards. All original app functions—that is, the article feed, first-person narratives, customizable reminders, local resources, community forum, and private chat function—remain organized as they were in the pilot study. A Positive Peers brand guide was created in the initial design phase by a professional marketing team to preserve PPA’s visual and content design. Informative articles are written by agency copywriters and study staff and are then vetted by clinicians. Participants are not directed or required to use the app in any specific way.

PPA is continuously monitored by a dedicated digital design team and PoPIT study staff. Conferences are held regularly to address issues identified by app administrators, reported by users, or noted by other study staff. The study coordinator relies on an electronic reporting system to alert the technical team, who respond promptly and effectively to app issues or bugs. PPA provides instructions for use, community standards, a privacy policy, and accessibility standards in an “About” tab within the app. All participants are made aware of data collection practices during informed consent. PPA does not sell or display advertising or rely on commercial sponsorships at this stage of development.

### Data Security and Accessibility

Existing infrastructure and security features of the PPA technology allow comprehensive and increasingly secure storage of PPA activity data. A centralized REDCap data management system is used to manage all study records and data. Unique modules are designed to securely store and track site recruitment, enrollment, informed consent and randomization, study questionnaires, qualitative interview participation, medical record review, EMAs, and study event reminders. All PPA usage data are stored on a secure, cloud-based, Health Insurance Portability and Accountability Act (HIPAA)– and System and Organization Controls 2 (SOC 2)–compliant server without any intrinsic ties to users’ demographic identities. Each study participant is assigned an ID upon enrollment, which is connected to their app and project activity. Questionnaire data are entered directly into and maintained in a secure REDCap database. PPA is compliant with WCAG for people with disabilities [73].

### Study Staff Training

The PoPIT study team delivers study training to staff at each collaborating site. Study staff at the trial sites reflect a range of expertise, with some highly trained in research support and others new to research work. The PoPIT training curriculum is organized into 3 parts: (1) orientation to the study, with emphasis on study staff roles and study flow (ie, prospective data collection); (2) specific instruction in the enrollment protocol, including review of study documents, consent, and randomization procedures; and (3) steps for downloading the PPA app and engaging with app functions. Staff new to research complete online basic research training through the host institution. PoPIT study instruction includes the formulation of a site-specific recruitment and promotion plan, as well as mock enrollment with PoPIT staff. The PoPIT study team conducts these trainings both in person and virtually, with an in-person session conducted at the site initiation visit. New site staff receive standardized onboarding in line with the training curriculum before actively enrolling participants in the study. Initial study training is supported by monthly site-specific videoconferencing meetings to reinforce the protocol and troubleshoot technical or other study-relevant problems. Tip sheets and notes supporting study procedures are maintained in a secure shared cloud storage system available to all study staff.

Site principal investigators and study staff meet monthly to discuss recruitment, best practices for patient enrollment, share successes and challenges, and troubleshoot issues. Concurrent with staff onboarding and site initiation, sites provide information to the PoPIT study team for integration into the PPA’s app infrastructure, including local contacts, logos, general information about the organization, and community resources that clinic staff wish to make readily available to their patient population. These resources include relevant medical clinics and contact information for local social service organizations. Many sites choose to include contacts for housing assistance, substance use and addiction support, mental health providers, food pantries, and support groups. These resources are transcribed into the app’s geographically organized resource database. All resources added to the database are independently verified by the PoPIT study team before being manually added to the app’s infrastructure.

### PPA Administrators

Each study site has a clinical study leader and a dedicated PPA administrator to carry out study activities. Some sites engage additional staff to support study-related activities such as recruitment, enrollment reporting, and medical record review. Study site PPA administrators receive separate training to maintain an online presence in the app that is welcoming but secondary to study participant interaction. For example, new participants are sent a welcome message by their site PPA administrator after entering the app. PPA administrators are asked to refrain from creating new posts in the community forum to avoid an administrator-dominated conversation, while commenting on posts when applicable. To date, as study enrollment has increased, PPA study app administrators have not initiated forum posts or discussions. Local PPA administrators are asked to maintain an unobtrusive presence in the app by introducing new users, providing occasional local or regional announcements, or engaging in private chats with participants to offer troubleshooting or technical support.

Generally, the PPA intervention is entirely user-directed and does not require additional human involvement. However, if the app is adopted as a clinical tool, a staff member at the clinic should be assigned to manage recruitment, registration, in-app support, and navigation services.

### Reminders and Notifications

The PoPIT study uses an automated alert and reminder system for follow-up questionnaire invitations. Reminders are keyed to each participant’s study enrollment date and study arm through the REDCap database system. Based on participant preferences, questionnaire invitations and reminders are sent via SMS text message or email. If contact information changes during the study, this is either reported directly by a participant to site staff or becomes apparent when a routine questionnaire invitation or reminder message is returned as undeliverable. Regular verification of contact information is emphasized to site staff as a critical component of project implementation and is continuously monitored. First, this routine outreach is an integral component of the PPA, functioning as an extension tool for clinical staff to promote engagement in care and support strategies outside the clinical encounter. Outside the trial environment, users receive notifications about updates and new features. Second, PPA includes a customizable notification system for the medication tracker, community forum, chat function, and event scheduling. Users can enable or disable these reminders and schedule the dates and times at which they are sent. Users may also opt in or out of notifications related to forum and chat activity. A third type of notification used in PPA is EMAs.

### Ecological Momentary Assessments

EMA is a method widely used to study behavior and mood in the settings in which they naturally occur [74,75]. Because of this immediacy, EMAs can support ecological validity by mitigating the memory limitations inherent in retrospective self-reports. Four general characteristics define EMAs: (1) data collection in real-world environments, (2) a focus on the individual’s current state of mind, (3) strategic selection of assessment moments, and (4) repeated assessments over time. Although the PPA records and maintains a log of each user action within the app, it remains unclear how users perceive the app while actively using it.

The PPA is premised on a user-centered model of digital media effects [59,61,63]. This approach suggests that users’ needs and the characteristics of the technology (ie, its affordances) interact to shape user engagement. Greater engagement with the mobile app is expected to influence self-efficacy in HIV management and increase the likelihood of favorable HIV clinical outcomes. As EMAs occur repeatedly in real time, they provide an opportunity to assess user engagement with the PPA more immediately than retrospective self-report. The goal of the EMA methodology is to link tracked physical interactions with the app (ie, natively recorded usage data) to users’ immediate perceptions of engagement in real time.

User engagement with interactive media comprises 4 validated attributes: (1) physical interaction, (2) interface assessment, (3) absorption, and (4) digital outreach [76]. Analyses indicate that greater physical interaction with the app and more positive assessments of the interface predict cognitive absorption with the content, which in turn is associated with stronger behavioral intentions to manage and socially share the content [76]. Our conceptual model has focused primarily on frequencies of physical interaction. EMAs can provide insight into user perceptions of the app interface and the extent to which users feel absorbed or attentive during use. We may also include a single-item measure of digital outreach in the form of the likelihood of recommending the app to others. Ultimately, we anticipate that increased user engagement will be associated with higher medication adherence, lower viral load, greater engagement in care, and reduced stigma. In this study, EMAs are delivered as SMS text messages sent to participants. The first EMA is deployed to all users 1 month after they begin using the app and focuses on physical interaction with the PPA (see Table 2). The second EMA is sent 2 months after app initiation and includes brief questions assessing current participant engagement with the PPA interface, as well as 4 items adapted from the System Usability Scale [77]. These micro-questionnaires serve as evaluative tools for assessing intervention implementation and effectiveness.

**Table 2:** EMA^a^ items at time 1 and time 2.

EMA items			Response option
**Time 1 EMA items: interaction with the PPA^b^**			
	Is the Positive Peers mobile app downloaded to your smartphone right now?	Yes/no	
	Did you use the Positive Peers mobile app today?	Yes/no	
	How many days in the last week did you use the Positive Peers mobile app?	Daily 4-6 days 2 or 3 days Once None	
	Did you take your medication yesterday?	Yes/no	
**Time 2 EMA items: interface assessment, absorption, and digital outreach**			
	When was the last time you used the Positive Peers app?	I haven’t used the Positive Peers app. Yesterday or today Within the last week Within the last month	
	In general, the app is interesting to me.	Strongly agree to strongly disagree	
	This app holds my interest.	Strongly agree to strongly disagree	
	While I was using the app, I was able to focus on the task at hand.	Strongly agree to strongly disagree	
	I would recommend this app to other people like me.	Yes/no	

^a^EMA: ecological momentary assessment.

^b^PPA: Positive Peers mobile app.

### Quantitative Data Collection and Measures

Data collection is planned for a total of 18 months for each participant. Three modes of data collection are used in the study. First, site staff collect medical record data, including date of diagnosis, HIV viral load, T-cell counts, and office visit dates, for the 12 months before enrollment and for 18 months after enrollment.

Second, participants receive an email or SMS text message notification to complete follow-up electronic questionnaires at 3, 6, 9, and 12 months after enrollment. Automated reminders are sent on the scheduled completion date; if the questionnaire is not completed, additional reminders are sent 1 week and 2 weeks later. If the questionnaire coincides with an office visit or cannot be completed remotely for any other reason, study staff can administer the pending questionnaire on site using a laptop or iPad (Apple Inc). Delayed-start participants are onboarded to the app, either in person or remotely by site staff, 6 months after enrollment. This group completes baseline, 3-month, and 6-month questionnaires before downloading and initiating app use.

Third, during the intervention period, EMAs are used to collect in-the-moment information about app use (eg, “Is the Positive Peers mobile app currently downloaded to your phone?”) and related perceptions. PPA provides a natural technological interface through which such perceptions or behaviors, and their correlates within the app or the broader social environment, can be assessed in a nuanced, dynamic, and longitudinal framework.

### Measures

#### Selection of Standardized and Validated Study Measures

Selected study measures make use of the PhenX toolkit [78], Patient-Reported Outcomes Measurement Information System (PROMIS) Toolbox [79,80], and Health Resources and Services Administration (HRSA) HIV/AIDS Bureau reporting standards [81]. Measures were selected based on our preliminary work, theoretical model, and relevance to the population (Table 3). The PhenX Toolkit is a searchable online database of recommended measurement protocols for human studies [82]. Database protocols are well established, broadly validated, reliable, and low burden for study participants. Measures for this study, derived from the PhenX toolkit, include a set of demographic characteristics and social determinants of health, as well as the Medical Outcomes Study Social Support Survey.

**Table 3:** PoPIT^a^ constructs, measures, and prospective data collection.

Construct	Measure	Collection
Participant characteristics [82]	PhenX: age, ethnicity and race, biological sex, sexual orientation, sexual identity, marital status, and educational attainment	Baseline^b^
Social determinants of health [83]	AAFP^c^ Social Needs Screening Tool: housing, food and transportation insecurity, utilities, employment, finances, and personal safety PhenX: internet access, access to health technology, and general social media use	Baseline and 12 months
Attitudes toward technology [84]	Social Media Usage & Attitudes Scale (MTUAS^d^)	Baseline, 6, and 12 months
Technology affordances [85]	Mobile Application Affordances Measure	3 and 6 months after app initiation
PPA^e^ engagement	Number of logins, duration (minutes), number of sessions, and engagement counts for each PPA function (articles, med tracker, resources, forum, and chat)	Concurrent with participant app use
Self-efficacy for managing chronic illness [86]	PROMIS^f^ subscales: managing emotions, social interactions, medication adherence, and general self-efficacy	Baseline, 3, 6, 9, and 12 months
Social support [87]	Medical Outcomes Study Subscales: emotional/informational support, tangible/instrumental support, and positive social interaction	Baseline, 3, 6, 9, and 12 months
Perceived HIV stigma [88]	HIV Stigma Scale subscales: personalized, disclosure, negative self-image, and public attitudes	Baseline, 3, 6, 9, and 12 months
Engagement in HIV care [89]	Pre- and postclinic visit dates and count	12 months before enrollment and 18 months after study completion
Viral suppression [89]	Preenrollment CD4 count and date Pre- and post-HIV viral load values	12 months before enrollment and 18 months after study completion

^a^PoPIT: Positive Peers Intervention Trial.

^b^Baseline data collection occurs at consent.

^c^AAFP: American Academy of Family Physicians

^d^MTUAS: Media and Technology Usage and Attitudes Scale.

^e^PPA: Positive Peers mobile app.

^f^PROMIS: Patient-Reported Outcomes Measurement Information System.

#### Participant Characteristics

Measured sociodemographic characteristics collected at baseline include age, ethnicity and race, biological sex, sexual orientation, sexual identity, marital status, and educational attainment. Measures were derived from the PhenX toolkit [82]. The toolkit measures are concise and widely used across health behavior studies involving marginalized groups.

#### Social Determinants of Health

The American Academy of Family Physicians developed an index of brief measures to serve as a screening tool for social determinants of health [83]. Measured social determinants of health include employment, housing, food and transportation insecurity, finances, and personal safety. The PhenX Toolkit provided measures of internet access, access to health technology, and general social media use.

#### Attitudes Toward Technology

The Media and Technology Usage and Attitudes Scale (MTUAS) [84] is a comprehensive 60-item measure designed to assess behaviors and attitudes across a wide variety of technologies and platforms. This study uses a version of the MTUAS attitudes subscale adapted to the PPA. This scale includes 12 five-point response items focused on measuring positive and negative attitudes toward technology use in general, as well as a third subscale that measures technology dependence. The subscales are reliable, and scores are significantly correlated with other measures of daily media use and technology-related anxiety.

#### Technology Affordances

Affordances are defined as features of user-technology interactions that offer gratification of perceived needs [85,89]. These features are independent of content and reflect how a user experiences a particular type of technology. Although more or less specific typologies of media affordances have been presented [62,63,90], this study relies on a set of 10 affordances (39 items) typical of social media use and tailored to mHealth app use. These include accessibility, bandwidth, social presence, privacy, network association, personalization, persistence, editability, conversation control, and anonymity. Items were adapted to PPA use, for example, “The Positive Peers app allows me to carefully craft my message before sending it” and “The Positive Peers app makes it seem like the other person is present,” and were set on a 7-point Likert response scale.

#### PPA Engagement

Following pilot study methods, intervention exposure is measured in terms of frequency of app use (number of logins and time spent) and engagement with its functions (eg, number of articles read, number of entries into a function, number of posts or replies) [17]. Participant intervention exposure is tracked natively in the PPA in real time and associated with the user’s study ID. Every screen tap for each app function is counted and aggregated in monthly reports. Community forum posts and replies are tracked verbatim, with time and date stamps. User data are separated from administrator and study staff data for evaluation using unique study IDs.

#### Self-Efficacy for Managing Chronic Conditions

Self-efficacy is measured in this study using the PROMIS instruments on self-efficacy for managing chronic conditions [91] and includes self-efficacy for managing (1) medications and treatments (4 items), (2) emotions (8 items), and (3) social interactions (8 items). Published validation efforts showed good concurrent validity and internal consistency [91]. Subscale items have 5 response options ranging from 1=I am not at all confident to 5=I am very confident.

#### Social Support

The PPA offers social support from others through the community forum and chat functions. Perceptions of social support will be tracked prospectively from baseline using the Medical Outcomes Study Social Support measure [87]. This measure is highly reliable and predictive of psychosocial outcomes across multiple chronic diseases [92,93].

This version includes 5-point Likert items that measure support across 3 dimensions: (1) emotional/informational support, (2) tangible/instrumental support, and (3) positive social interaction (ie, companionship). A fourth subscale, affectionate support, was beyond the scope of this study and was not included in the questionnaires.

### Outcomes

#### Perceived HIV Stigma

The 12-item (short version) Perceived HIV Stigma Scale is used to measure overall perceived stigma and distinct dimensions (3 items each), including (1) personalized stigma, (2) disclosure concerns, (3) concerns about public attitudes, and (4) negative self-image. A 4-point Likert response format assesses each domain. Scores can be summed or averaged for each 3-item subscale and across all 12 items to yield a total stigma score, with higher scores indicating greater perceived stigma. Psychometric evaluation in a national sample of adults with HIV supported a 4-factor structure with good fit and acceptable internal consistency across subscales.

#### Clinical Outcomes

Retention in HIV care and viral suppression are measured using standardized performance measures developed by the HRSA/HIV/AIDS Bureau [89]. Measures selected for this study are retention in HIV medical care (2 or more visits separated by at least 90 days for HIV care within the measurement year) and HIV load suppression (the most recent viral load <200 copies/mL during the measurement year). Values for outcome variables (engagement in care and viral suppression) are derived from a retrospective medical record review conducted by site study staff using a study-specific form. Engagement in care is determined by at least one office visit with an HIV clinician per 6-month interval for 12 months before and 6 months after study enrollment. Viral suppression is determined by assessing the participant’s viral load, as noted in laboratory values, typically required at least every 6 months per the standard of care. A value of <200 copies/mL is considered suppressed. Values for each variable are established for the year before study enrollment, at baseline enrollment, and then at 6-month intervals for 18 months.

### Power Analysis

Group sample sizes of 106 in the Positive Peer group and 106 in the control group achieve 90% power to detect a difference between group proportions of 0.20. This is the minimum sample size required in each group to detect the desired difference in proportions. The proportion in group 1 (the treatment group) is assumed to be 0.60 under the null hypothesis and 0.80 under the alternative hypothesis. The proportion in group 2 (the control group) is 0.60. The test statistic used is the 2-sided *Z* test with pooled variance. The significance level of the test was set at .05. Several assumptions regarding baseline viral suppression were tested based on the literature. The current sample size is based on 63% viral suppression at baseline and a planned 80% in the intervention group. If baseline viral suppression is lower, a smaller sample size would be needed to demonstrate a 20% improvement in viral suppression. Based on this estimation, we aim to recruit and randomize 250 eligible individuals to the study from 6 clinic sites over 24 months, allowing for potential loss to follow-up. It is important to note that patients without documentation of a clinic visit during the assessment period are designated as out of care and will be assumed to be HIV virally unsuppressed. Available data on other outcomes and covariates, however, will be handled using mixed-effects models, which employ maximum likelihood estimation and assume missing-at-random patterns to support valid inferences.

### Planned Statistical Analysis

Aims 1 and 2 entail data collection and analysis to provide evidence of PPA effectiveness. Outcomes include retention in HIV care (proportion of enrolled patients with laboratory results in each 6-month period of the study), HIV load suppression (proportion of enrolled patients whose viral load measurement at 6 months in the study is <200 copies/mL), as well as self-reported psychosocial variables, specifically perceived technology affordances, attitudes toward technology, and self-efficacy for managing chronic conditions. We expect that key demographics, technology covariates, and PPA use will predict self-efficacy, which will be directly associated with (1) retention in care, (2) viral suppression, and (3) HIV-related perceived stigma. Additionally, we plan to test potential covariate mediation and examine theoretical linkages in our model of PPA effects.

Analyses of repeated measures will be performed using generalized linear mixed-effects models with a logit link function for binary outcomes (ie, viral suppression and engagement in care) and linear mixed-effects models for continuous outcomes (eg, perceived stigma, resilience). Time will be treated as a fixed, repeated factor within patients, and patient-specific random effects will be included to account for within-patient correlation. To appropriately model the hierarchical structure of observations nested within patients and patients nested within sites, the site will be included as a random effect, providing an estimate of between-site variability. Multilevel mediation models will be used to evaluate potential mediating effects of covariates. *P* values will be adjusted using Bonferroni or Benjamini-Hochberg correction procedures to control the family-wise type I error rate due to multiple comparisons across distinct psychological outcomes.

### Qualitative Data Collection

#### Longitudinal Semistructured Interviews and Interpretive Thematic Analysis

In-depth semistructured interviews with participants explore experiences with PPA initiation and use, including participants’ reflections on joining a mobile app community focused on HIV care. Interviews are conducted longitudinally at approximately 3, 7, and 11 months after initiating use within both immediate- and delayed-start cohorts, with largely independent samples of 8-10 participants from each cohort at each time point. Interviews explore how participants learn, navigate, and maintain PPA use, focusing on perceived benefits, costs, and motivations for ongoing engagement. Anchoring the convergence of mixed methods, interviews also elicit reflections on YPWH’s experiences with the study’s measured quantitative constructs (eg, experienced social needs that thwart or facilitate engagement in HIV care, perspectives and attitudes toward social media use, perceived self-efficacy for health management).

For sampling and analysis, participants are selected purposively to balance participation across key demographic strata of interest (eg, racial and ethnic identity, gender identity). All interviews are professionally transcribed verbatim and analyzed using NVivo (Lumivero) software with an interpretive, phenomenologically informed approach [94] and a constant comparative approach [95] to thematic analysis. Our approach to analysis is phenomenologically informed in that study participants share a core set of experiences related to their identities as YPWH. To enhance rigor and validity, a subgroup of 5 YPWH participants will serve as thematic auditors and critically respond to qualitative findings and interpretations.

#### Qualitative Analysis

In support of aims 2 and 3, interviews with a subset of the delayed- and immediate-start cohorts will focus on YPWH’s experiences with initiating and continuing PPA use, as well as any thoughts or concerns participants may have had about joining a mobile app community focused on HIV care. Anchoring the convergence of methods, interviews will also elicit YPWH’s reflections on their experiences with the study’s measured quantitative constructs (eg, experienced social needs that thwart or facilitate engagement in HIV care, perspectives and attitudes toward social media use, perceived self-efficacy for health management). Interviews will be conducted 2-4, 6-8, and 10-12 months after initiating use. These interviews will explore YPWH’s experiences with learning, navigating, and maintaining PPA intervention use; for example, interviews will focus on eliciting how YPWH make sense of perceived benefits or costs of intervention use and what personal motivations facilitate ongoing engagement.

Participants from both cohorts will be asked to reflect on and compare their thoughts and preferences defined before engaging with the PPA intervention. Approximately 40 participants across the 6 clinic sites will be selected, stratified by immediate- (n=20) and delayed-start (n=20) intervention engagement. Based on prior research in similar phenomenological investigations of clinical interventions, thematic saturation is expected to be reached with interviews involving between 8 and 20 participants. Enrollment will continue until thematic saturation is achieved (ie, until no new thematic insights are identified). Qualitative participants will be selected purposively to ensure balanced participation across demographic strata of interest (eg, racial and ethnic identity, socioeconomic position). All interviews will be professionally transcribed verbatim.

Interviews will be coded using NVivo qualitative data analysis software. Our approach to analysis is phenomenologically informed, as study participants share a core set of experiences related to their identities as YPWH. Given the goal of convergent interpretation with quantitative study results, a thematic constant comparative approach will be used to analyze transcripts. Analysis will consist of thematic organization and concept clustering, conducted to illuminate participants’ health maintenance preferences and their holistic understanding and experience of the measured outcomes.

A mixed methods convergence matrix will further clarify the synthesis, overlap, and divergence of key study findings and will provide a validity check. The interview guide, informed by the perspectives of patients in the demonstration pilot, will be refined before study initiation and following the initial rounds of interviews. Interviews will be conducted via secure videoconferencing. Two site personnel will serve as thematic auditors to critically respond to qualitative findings and interpretations. Reporting of qualitative and mixed methods findings will adhere to the COREQ (Consolidated Criteria for Reporting Qualitative Research) guidelines [96].

### Ethical Considerations

This study is being conducted in accordance with guidelines outlined in the Declaration of Helsinki and the Common Rule [97]. The PoPIT protocol was approved on October 26, 2023, by the MetroHealth System IRB (Protocol: CR00001226), and a reliance agreement was established with each of the academic sites. The 2 federally qualified health centers do not have their own IRB and deferred to the MetroHealth IRB. Informed consent is completed in person at the enrollment visit.

## Results

The research protocol (1R01MD019185-01) was funded for US $903,363.00 in direct costs by the National Institute for Minority Health and Health Disparities on September 24, 2023, for 5 years. The study began recruiting patients on June 3, 2024, and will continue accrual until November 27, 2026.

## Discussion

### Anticipated Findings

Mobile apps to support chronic disease management are appealing, and evidence for their efficacy is building [35,98,99]. Social networking technologies offer quick and constant access to social ties and create diverse virtual social networks, despite potentially discriminating social factors such as age or race [100,101]. These virtual interpersonal networks allow users the opportunity to observe and create norms for positive self-care behaviors, including illness self-management and social support [50,58,100].

The goal of this project is to evaluate the PPA as a clinic-based tool for helping patients access peer support, remain in HIV care, and sustain viral suppression. Although traditional models of patient navigation can be effective for PWH in care [102,103], navigation augmented with tracking tools and a constant source of diverse, relevant information paired with peer experience can (1) rapidly build trust, (2) demystify HIV care complexity, and (3) build self-efficacy toward independent self-care. HIV clinic or nonprofit staff can use the PPA to offer navigation services ranging from medication and viral load testing reminders to peer support and assistance with accessing local living resources. Study results will determine whether this tool helps organizations and clinics reach the Ending the Epidemic goals. Furthermore, data will inform optimal deployment of this mHealth tool for newly diagnosed and reengaging PWH.

Concerns about mHealth scale-up suggest a gap between what researchers consider meaningful and appropriate and what target users find relevant [104,105]. Some social media–based interventions have relied on generic commercial platforms adapted to a disease with little input from end users [106,107]. The PPA was conceived and continues to be shaped by YPWH. Formative app development included user input and decision-making for most app features, content, and imagery, including the look of the app (eg, avatars, backgrounds, badges), article topics, resource needs, and the arrangement of features and functions [17]. The PPA offers a decentralized pathway to care engagement grounded in peer solidarity rather than structured, top-down case management approaches [108]. Clear evidence of effectiveness will allow us to expand PPA availability to new regions and infuse vitality and diversity into the existing PPA social network.

### Conclusions

The PPA offers a mobile social network that provides instrumental, emotional, and tangible support in a space where the stigma of HIV status is neutralized. Validation of the PPA as a clinical support tool may provide underresourced clinics with a relevant resource for augmenting outreach and psychosocial support. Completion of these aims will contribute to a national partnership among geographically distinct providers and their patients. Broad dissemination, award-winning creative content, and secure, stable, and responsive tracking tools create an unprecedented opportunity to improve HIV outcomes for high-risk and disparity populations of YPWH.

## Appendix

Multimedia Appendix 1 Peer review report from the ZMD1 IN (A1) - National Institute on Minority Health and Health Disparities Special Emphasis Panel, Promoting Viral Suppression and HIV Prevention Interventions Amongst Health Disparity Populations (National Institutes of Health, USA).

## Abbreviations

COREQ: Consolidated Criteria for Reporting Qualitative Research
EMA: ecological momentary assessment
HIPAA: Health Insurance Portability and Accountability Act
HRSA: Health Resources and Services Administration
IRB: institutional review board
mHealth: mobile health
MTUAS: Media and Technology Usage and Attitudes Scale
PoPIT: Positive Peers Intervention Trial
PPA: Positive Peers mobile app
PRISM: Practical Robust Implementation and Sustainability Model
PROMIS: Patient-Reported Outcomes Measurement Information System
PWH: people with HIV
RE-AIM: Reach, Effectiveness, Adoption, Implementation, and Maintenance
SNT: Social Networking Theory
SOC 2: System and Organization Controls 2
WCAG: Web Content Accessibility Guidelines
YPWH: young people with HIV
