# Supplementary material for: Evaluation of the Positive Peers Mobile App for Supporting the Viral Suppression of Young People With HIV: Protocol for a Concurrent Mixed Methods Evaluation With Randomized Controlled Clinical Trial and Observational Cohort
Source: JMIR Res Protoc. 2026 Mar 17;15:e87601. doi: 10.2196/87601 (PMC13147924; doi:10.2196/87601)
Supplement: Multimedia Appendix 1 [file resprot_v15i1e87601_app1.pdf]

SUMMARY STATEMENT
(Privileged Communication)

PROGRAM CONTACT:
JARRETT Johnson
301-402-1366
johnsonjara@mail.nih.gov

Release Date: 07/12/2023
Revised Date:

Application Number: 1R01MD019185-01

Principal Investigators (Listed Alphabetically):

AVERY, ANN K (Contact)
STEP, MARY M.

Applicant Organization: CASE WESTERN RESERVE UNIVERSITY

Review Group: ZMD1 IN (A1)
National Institute on Minority Health and Health Disparities Special Emphasis Panel
Promoting Viral Suppression and HIV Prevention Interventions Amongst Health Disparity Populations
AIDS

Meeting Date: 06/29/2023
Council: AUG 2023
Requested Start: 09/01/2023
Opportunity Number: RFA-MD-23-007
PCC: CPS10

Project Title: Randomized Control Trial of Positive Peers mHealth app as a clinic-based intervention to optimize HIV outcomes among young, minority persons living with HIV
SRG Action: Impact Score:49
Next Steps: Visit https://grants.nih.gov/grants/next_steps.htm
Human Subjects: 48-At time of award, restrictions will apply
Animal Subjects: 10-No live vertebrate animals involved for competing appl.
Gender: 1A-Both genders, scientifically acceptable
Minority: 1A-Minorities and non-minorities, scientifically acceptable
Age: 7U-Only Adults, scientifically unacceptable

Project Year	Direct Costs Requested	Estimated Total Cost
1	748,392	1,153,673
2	749,815	1,155,866
3	749,238	1,154,977
4	749,098	1,154,761
5	749,929	1,156,042
TOTAL	3,746,472	5,775,319

NEW INVESTIGATOR

1R01MD019185-01 AVERY, ANN

PROTECTION OF HUMAN SUBJECTS UNACCEPTABLE
INCLUSION ACROSS THE LIFESPAN: UNACCEPTABLE

RESUME AND SUMMARY OF DISCUSSION: This application is submitted in response to RFA-MD-23-007, “Promoting Viral Suppression among Individuals from Health Disparity Populations Engaged in HIV Care (R01)” by Case Western Reserve University with Drs. Ann Avery and Mary Step as MPI. The Investigators propose to evaluate the effectiveness of a Positive Peers App in six *End the HIV epidemic* (EHE) areas using clinics to reach racially and/or sexually minoritized young living with HIV. Preliminary studies have shown the intervention to be acceptable and effective, particularly among younger participants, with promising results for improving viral suppression. This proposed study expands the study population to multiple EHE sites around the country, although the recruitment inclusion minimal age has been restricted to subjects 18 years old. The review panel members noted the strong preliminary data. The application is significant and timely given that HIV prevalence is significantly higher, and viral suppression rates are lower in racially and/or sexually minoritized young living with HIV. The application builds upon strong community engagement. The research team is excellent and well-integrated, and the environment is adequate for the proposed work. However, major weaknesses are also noted: The intended approach to enroll subjects of ages 18-30 departs from supportive preliminary data and may limit the significance of the intervention. There are uncertainties about fidelity of the app use over time and how this might affect sustained outcomes. It is unclear whether a peer will be available 24/7, and how the support across the different clinical sites/states will be standardized. Gamification seems to be limited in the app. It is unclear how the app will grab the attention of participants who are unmotivated. The analysis plan for Reach seems to be missing. Cost is one variable of maintenance, but it is unclear how it will be analyzed and what will be considered a reasonable/sustainable cost. There are concerns about user privacy with pop ups and phone log-in process. It is unclear how stigma is targeted and reduced in the app. There seems to be a poor integration of the HIV clinic staff into the design and implementation of the study. Not all the letters of support from the six study sites have been obtained. Overall, the review panel scored this application as good, with potential for moderate impact in the field.

DESCRIPTION (provided by applicant): The Positive Peers mobile app is an original platform developed by and for the hardest to reach HIV disparity populations, young people with HIV who identify as racial, ethnic and/or sexual/ gender minorities. This app holds potential to provide extensive, customizable, self- management tools (i.e., wellness tracker, community forum, chat, frequent original blogs) to young people with HIV anywhere in the US. The Positive Peers app provides health information, health management tools and virtual community support. While the app itself offers a safe place for young persons with HIV to get health information and support, it's use is enhanced by the presence of local peer administrators who provide navigation, support and coaching to users. The proposed study seeks to evaluate its effectiveness in improving viral suppression among minority disparity populations 18-30 years of age who are either newly diagnosed, out of care or not virally suppressed using a randomized control trial design supplemented by an observational cohort of persons who decline to use the app. Clinics in six high priority Ending the HIV epidemic jurisdictions will train staff as app administrators and utilize the app as a clinic- based tool. Our primary objective is to improve HIV outcomes by offering peer interaction, targeted retention and adherence messaging, and interactive trackers and reminders in one smartphone app. Our specific aims are: Aim 1: Compare the effectiveness of HIV care supported by the PPA to usual care for retention in HIV care and viral suppression of newly diagnosed or re-engaged high priority younger adults with HIV. Aim 2: To identify factors that predict user engagement with primary PPA components and associated effects on retention in care, viral suppression, and HIV related perceived stigma. Aim 3: To determine intervention adoption,

usability, fidelity, and cost across study sites. These aims will be addressed in a parallel cohort design randomized controlled trial of 250 newly diagnosed or out of care YPWH from designated high priority sites. Participants will be allocated 1:1 to receive the PPA app upon study entry or to a delayed intervention arm where they will receive the usual care with attention controls for 6 months. This will allow for effectiveness evaluation during the earliest phase of adjustment to the diagnosis while also allowing for longitudinal outcome effects.

PUBLIC HEALTH RELEVANCE
This research aims to test the effectiveness of the Positive Peers smartphone application, a navigation and support tool targeted to HIV disparity populations in a randomized clinical trial at six high priority clinical sites. The app combines access to an established peer support community and vital medical management tools with skilled clinic-based navigation. Data gained from this study will provide clinics a specialized and freely available tool for improving retention in care and viral suppression in their minority patient populations.

CRITIQUE 1

Significance: 2
Investigator(s): 3
Innovation: 3
Approach: 5
Environment: 1

Overall Impact: This is an interesting application from a team that has developed an app designed to support young people who are newly enrolled in HIV care and treatment, those who have dropped out of care, and those who are not virally suppressed. The app uses some gamification, although not as much as maybe other apps have, peer support, and information to support adherence in care. The app targeted younger individuals (people aged 13-30 years old). Primarily Black and Hispanic MSM have used the app in the pilot phase, the appropriate target population. The app appears to be effective in targeting behavior change (adherence and viral suppression) through education, peer support (both from other patients and official peer-educators) and providing reminders and awards for taking medication. Preliminary studies have shown the intervention to be acceptable and effective, particularly among younger participants. This proposed study expands the study population to multiple EHE sites around the country (LOSs suggest strong enthusiasm for the app use) and enrollment targets are feasible given the HIV incidence at each location. The contact PI (Avery) developed the app and conducted the original studies with the app. She also has a team working to keep it updated and relevant. Dr. Step and Dr. Avery have worked together for many years to evaluate the Positive Peers app. But neither seem to have much experience leading NIH funding. Enthusiasm is reduced due to a few aspects about the app that appear confusing (e.g., is a peer available 24/7) and some confusion with implementation outcomes and analysis plans in Aim 3. Specifically, the costing component is under explored and the concept of adoption is being inappropriately applied/measured. Despite these small issues, the overall application is strong and has the potential to be highly impactful.

1. Significance:

Strengths

- The app has been developed and has a long history of being acceptable to the populations of interest (Young people living with HIV (YPLWH) are very likely to be at the intersection of

multiple marginalized identities and disenfranchised communities - LGBTQIA (Lesbian, Gay, Bisexual, Transgender, Queer, Intersex, Asexual), BIPOC (Black, Indigenous, People of Color).

- HIV prevalence is significantly higher in the populations that are being addressed and viral suppression rates are lower.
- Finding a mechanism to provide social support to groups who feel isolated can improve treatment and mental health outcomes.

Weaknesses

- There are now a number of apps available to support adherence. How is this one different?

2. Investigator(s):

Strengths

- The contact PI (Avery) developed the app and conducted the original studies with the app. She also has a team working to keep it updated and relevant.
- Dr. Step and Dr. Avery have worked together for many years to evaluate the Positive Peers app.
- Dr. Step has the experience to support the qualitative components of the project.

Weaknesses

- Dr. Step does not appear to have any experience leading an NIH funded project and while Dr. Avery leads a Core and is a co-I on several grants she does not have substantial NIH funding.

3. Innovation:

Strengths

- Delivering peer support via an app is both appropriate and innovative for this age group.

Weaknesses

- There is not much in terms of gamification in the app, which seems to be the way these apps are moving.
- Not clear how the app will grab the attention of participants who are unmotivated.

4. Approach:

Strengths

- The team has identified key EHE jurisdictions to study the app, has support from facilities in each, and understands the data sufficiently to design the study.
- There is strong preliminary data to show that the app has a positive impact on treatment adherence and viral suppression.
- The provision of peer support through the app will be provided at a key time in these young people's lives- both when they are young and when they are either just diagnosed or are struggling to be adherent.
- The app provides feedback – light gamification- in the way of badges for adherence to treatment.

- The team has mapped the Positive Peers functions onto the NIMHD disparities research framework and matches well with the needs of this population.

Weaknesses

- The comparison group (standard of care) may vary substantially by state/clinic.
- I'm not clear how the person-to-person navigation can function at any time. Do you have people employed 24/7 to support these activities? How will you standardize support across the different clinical sites/states?
- People who are newly diagnosed feel like they are quite different than those who have dropped out of care or who are not virally suppressed.
- The use of RE-AIM is not in following with the original design. The analysis plan for Reach is not described. The authors are assessing adoption as a patient level variable when it should be a facility/provider variable. Cost is one variable of maintenance, but I don't have a sense of how it will be analyzed or what will be considered a reasonable/sustainable cost.
- It would be nice to have additional data on what the app offers in each category- examples of article topics or questions that people ask using the messaging system.
- Is there concern that the cluster-style recruitment will necessitate a clustered analysis?

5. Environment:

Strengths

- Very supportive environment from the universities to the health facilities.

Weaknesses

- None noted.

Study Timeline:

Strengths

- None noted.

Weaknesses

- It would be nice to have additional details about enrollment targets by time period, the types of conferences and journals that will be targeted, etc.

Protections for Human Subjects

Acceptable Risks and/or Adequate Protections

- adequate projections for those under 18 years of age

Data and Safety Monitoring Plan (Applicable for Clinical Trials Only):

Acceptable

- The team will track app activity and have a reasonable plan for data storage and monitoring the safety of participants.

Inclusion Plans

- Sex/Gender: Distribution justified scientifically.
- Race/Ethnicity: Distribution justified scientifically.
- For NIH-Defined Phase III trials, Plans for valid design and analysis: Scientifically acceptable.
- Inclusion/Exclusion Based on Age: Distribution justified scientifically.

Vertebrate Animals
Not Applicable (No Vertebrate Animals)

Biohazards
Not Applicable (No Biohazards)

Applications from Foreign Organizations
Not Applicable (No Foreign Organizations)

Select Agents
Not Applicable (No Select Agents)

Authentication of Key Biological and/or Chemical Resources
Not Applicable (No Relevant Resources)

Budget and Period of Support
Recommend as Requested

- PI effort is reasonable, may need additional support to the sites for the peer navigators.

CRITIQUE 2

Significance: 4
Investigator(s): 4
Innovation: 4
Approach: 7
Environment: 1

Overall Impact: This study aims to evaluate the effectiveness of a Positive Peers App in six EHE areas using clinics to reach racially and/or sexually minoritized youth ages 13-24. The PPA is currently offered to patients in Ryan White programs and has promising results for improving viral suppression. Strengths of the proposal are the strong preliminary data, however there are some concerns about fidelity of the app use over time and how this might affect sustained outcomes.

1. Significance:

Strengths

- The population that PPA is created for and by is a high-risk, high-need population with few interventions that are context specific for them.

Weaknesses

- App usage wanes over time. Thus, the utility of the app and relevant outcomes that the app targets may not be sustained.

2. Investigator(s):

Strengths

- PI and co-I have a strong, existing relationship on the proposed app.

Weaknesses

- No one on the study team identified with expertise in EMA assessments.

3. Innovation:

Strengths

- Integration of theoretical frameworks novel public health is a strength.
- Understanding user engagement is novel.

Weaknesses

- Patient navigation and directing services to populations with the highest need are not innovative strategies.

4. Approach:

Strengths

- There is strong community engagement. Specifically, the app is built by and for the population of interest.
- Really good preliminary data on both health outcomes and app usage.
- Data collection measures are strong and well-conceived.
- The cost analyses are a strength that will help understand scalability and sustainability of the intervention.

Weaknesses

- There is some concern about user privacy with pop ups on the ad and how this may “out” a person LWH if someone has their phone or they are opening their phone by someone.
- Also, privacy concerns about the log-in. Does the app stay logged in on the phone? Does the user have to manually do this every time they want to use the app? Is it a dual step process?
- No implementation science framework is provided.
- Unclear how stigma is targeted and reduced in the app.
- Each site needs a PPA and a clinical leader. From the grant perspective, the investigative team doesn’t seem built up for this. From a pragmatic perspective, this may be infeasible in a real-

world setting. How realistic is it that a clinic would use this outside of a clinic visit where they would not be able to bill?
- Who will perform randomization in the clinic?
- It is unclear what the EMA assessments will add. The participant burden of this seems high and is uncompensated to the participant.
- There needs to be more justification of the delayed start group. In the current analytic plan, there is no hypothesis to examine whether six versus 12-months of using the app relates to adherence, so essentially the delayed start group is similar to the observational cohort. The benefits of randomization are lost because the analysis is ITT. So, it is unclear what this group adds.
- The implementation metrics of adoption should also examine clinic level outcomes.
- It is unclear how retention and fidelity across study sites will be achieved.
- Qualitative interviews to understand individuals who do not engage with the app would be useful. Similarly, those who use the app and achieve suppression/ don’t achieve suppression would also be useful.
- Clinic level factors may additionally affect participant retention and adherence. It is unclear how these will be accounted for.
- There is strong concern that letters of support from all of the study sites have not been obtained.

5. Environment:

Strengths
- Metrohealth has a strong research environment.

Weaknesses
- None noted.

Study Timeline:

Strengths
- Acceptable timeline.

Weaknesses
- None noted.

Protections for Human Subjects

Unacceptable Risks and/or Inadequate Protections
- There are some privacy concerns about medication alerts popping up on participants' phones.

Data and Safety Monitoring Plan (Applicable for Clinical Trials Only):

Acceptable

Inclusion Plans
- Sex/Gender: Distribution justified scientifically.

- Race/Ethnicity: Distribution justified scientifically.
- For NIH-Defined Phase III trials, Plans for valid design and analysis: Not applicable.
- Inclusion/Exclusion Based on Age: Distribution justified scientifically.

Vertebrate Animals
Not Applicable (No Vertebrate Animals)

Biohazards
Not Applicable (No Biohazards)

Applications from Foreign Organizations
Not Applicable (No Foreign Organizations)

Select Agents
Not Applicable (No Select Agents)

Resource Sharing Plans
Not Applicable (No Relevant Resources)

Authentication of Key Biological and/or Chemical Resources
Not Applicable (No Relevant Resources)

Budget and Period of Support
Recommend as Requested

CRITIQUE 3

Significance: 2
Investigator(s): 3
Innovation: 3
Approach: 4
Environment: 1

Overall Impact: Technology-based interventions are likely going to serve as a major component of HIV care and prevention in the near future, and this is a promising app with a good history of empirical support. However, there is underwhelming integration of the HIV clinic staff into the design and implementation of the study. Further, I am unconvinced by the rationale to exclude persons under the age of 18 because of a policy in their “chat” function. I would prefer a design that includes persons who could benefit most from the app (including those aged 13-17), and which does more than just give lip service to the HIV clinic staff that will be integral to the operation of this study.

1. Significance:

Strengths

- Excellent engagement with multiple EHE geographic hotspots.
- Young MSM and trans women of color are extremely high-priority EHE populations.

Weaknesses

- Though HIV-related stigma plays a big role in the analytical plan (and in the lives of the proposed participant populations), little is done to unpack the nuanced and specific ways HIV-related stigmas influence the behavior of young persons, MSM, and/or trans women of color. The likelihood of the app's success depends in part on its ability to navigate these specific and varied issues, and yet little is done to demonstrate understanding of the issues at hand.

2. Investigator(s):

Strengths

- Excellent team to perform an evaluation of the PPA.

Weaknesses

- Underwhelming level of input from persons working directly in the HIV clinics in which this is to be applied; design largely matches that of a standard academic or research institute-funded study.

3. Innovation:

Strengths

- Wide and diverse range of content availability, created and curated by focal population members.
- Leveraging an engaging mHealth app for patient navigation purposes in-clinic.

Weaknesses

- Knowledge and review of media theories of technology use are not in-and-of-themselves innovations related to this project.

4. Approach:

Strengths

- The delayed-start design is a good way to maximize data, cost efficiency, and participant care.
- Broad engagement with EHE geographic hotspots minimizes worry of narrow or contextual effects.
- Use of EMA for granular understanding of participants' reactions to the app experience.
- Mixed method design increases chances of finding meaningful and interpretable results.
- Application of RE-AIM framework for process evaluation.

Weaknesses

- The choice to enroll 18–30-year-old participants is unusual, given the need is more dire among 13-24 year olds (the original target population for the PPA).
- The “multilevel” nature of this design is questionable. Aside from an attractive table hypothesizing effects at various levels that hypothetically could result from app use, there is little attempt to influence or measure effects at multiple levels (i.e., individual, interpersonal, environmental).
- The actual analytic utility of the prospective observational cohort is unclear, given that the rate at which it will accrue members vs. the randomly assigned groups is unclear. I might prefer to see less weight included on the observational cohort, perhaps moving comparisons to the group to a secondary aim.
- The power analysis section is underwhelming and would be improved by a deeper discussion of specific analyses, the potential for varying amounts of attrition (and its effect on power), and a more precise set of starting conditions (e.g., 63% instead of 60%).

5. Environment:

Strengths

- Excellent clinical sites, broad representation across EHE sites.
- Strong research-related resources available across sites.

Weaknesses

- None noted.

Study Timeline:

Strengths

- Timeline seems feasible and appropriate, given prior experience and success.

Weaknesses

- Lack of conference presentations/dissemination.

Protections for Human Subjects

Acceptable Risks and/or Adequate Protections

- I would have liked to see the rules and guidelines for app engagement, as well as more about how frequent violations to the "chat" rules of behavior are, and what "punishments" have been carried out. I'd also like to know if there were any deleterious knock-on effects from things that may have been said in "chat" that violated the terms of use. Depending on these answers, Human Subject Protections may need to be updated.

Data and Safety Monitoring Plan (Applicable for Clinical Trials Only):

Acceptable

- I would have preferred a DSMB meeting every 6 months for the first year, and then annually after that if the DSMB agrees.

Inclusion Plans

- Sex/Gender: Distribution justified scientifically.
- Race/Ethnicity: Distribution justified scientifically.
- For NIH-Defined Phase III trials, Plans for valid design and analysis:
- Inclusion/Exclusion Based on Age: Distribution not justified scientifically.

They make it clear in their review of epidemiologic literature that the group of primary interest is probably actually aged 13-24. I don't see that they made a convincing point that their intended approach to enroll ages 18-30 is the correct one (especially since PP has previously been applied among populations as young as 13). If the "chat" function needs to be updated to segment persons by ages, or to have better screening and monitoring in order to allow minors to engage with it, then I would suggest making those changes. There are countless apps available that have chat features which minors use, I don't see this as a sufficient obstacle to impede the science. If not, then I would suggest removing the "chat" function and making the app available to those who need it.

Vertebrate Animals

Not Applicable (No Vertebrate Animals)

Biohazards

Not Applicable (No Biohazards)

Applications from Foreign Organizations

Not Applicable (No Foreign Organizations)

Select Agents

Not Applicable (No Select Agents)

Authentication of Key Biological and/or Chemical Resources

Not Applicable (No Relevant Resources)

Budget and Period of Support

Recommend as Requested

THE FOLLOWING SECTIONS WERE PREPARED BY THE SCIENTIFIC REVIEW OFFICER TO SUMMARIZE THE OUTCOME OF DISCUSSIONS OF THE REVIEW COMMITTEE, OR REVIEWERS' WRITTEN CRITIQUES, ON THE FOLLOWING ISSUES:

PROTECTION OF HUMAN SUBJECTS: UNACCEPTABLE

- Privacy concerns about medication alerts popping up on participants' phones.

INCLUSION OF WOMEN PLAN: ACCEPTABLE

INCLUSION OF MINORITIES PLAN: ACCEPTABLE

INCLUSION ACROSS THE LIFESPAN: UNACCEPTABLE

- Arguably the group of primary interest may be 13-24. The intended approach to enroll ages 18-30 is not well justified.

COMMITTEE BUDGET RECOMMENDATIONS: The budget was recommended as requested.

Footnotes for 1R01MD019185-01; PI Name: AVERY, ANN K

NIH has modified its policy regarding the receipt of resubmissions (amended applications).See Guide Notice NOT-OD-18-197 at https://grants.nih.gov/grants/guide/notice-files/NOT-OD-18-197.html. The impact/priority score is calculated after discussion of an application by averaging the overall scores (1-9) given by all voting reviewers on the committee and multiplying by 10. The criterion scores are submitted prior to the meeting by the individual reviewers assigned to an application, and are not discussed specifically at the review meeting or calculated into the overall impact score. Some applications also receive a percentile ranking. For details on the review process, see http://grants.nih.gov/grants/peer_review_process.htm#scoring.

MEETING ROSTER

The roster for this review meeting is displayed as an aggregated roster that includes reviewers from multiple MD Special Emphasis Panels Meetings
for the 2023/08 council round.
This roster for MD is available here.
